# Development of Dispersive Liquid-Liquid Microextraction Procedure for Trace Determination of Malathion Pesticide in Urine Samples

**Published:** 2019-10

**Authors:** Maryam RAMIN, Monireh KHADEM, Fariborz OMIDI, Mehran POURHOSEIN, Farideh GOLBABAEI, Seyed Jamaleddin SHAHTAHERI

**Affiliations:** 1. Department of Occupational Health Engineering, School of Public Health, Tehran University of Medical Sciences, Tehran, Iran; 2. Research Center for Environmental Determinants of Health (RCEDH), Health Institute, Kermanshah University of Medical Sciences, Kermanshah, Iran; 3. Institute for Environmental Research, Tehran University of Medical Sciences, Tehran, Iran

**Keywords:** Malathion, Dispersive liquid-liquid microextraction, One variable at a time, Urine, HPLC

## Abstract

**Background:**

Measurement of pesticides in biological matrices is become a serious challenge for researches because of their very low concentration in different matrices. The aim of this study was to develop a new sample preparation method with high accuracy and validity, simplicity and short retention time for determination of malathion.

**Methods:**

Dispersive liquid-liquid micro-extraction (DLLME) technique coupled with high-performance liquid chromatography equipped with ultraviolet detector (HPLC-UV) developed for trace extraction and determination of malathion pesticide in human urine samples. This study was done in 2017 at Tehran University of Medical Sciences, Tehran, Iran. One variable at a time (OVAT) method was used to optimize parameters affecting the malathion extraction. Different parameters such as extraction solvent, disperser solvent, and volume of the extraction solvent, volume of the disperser solvent, centrifugation time and speed, salt addition, and sample pH were studied and optimized.

**Results:**

Under the optimized conditions, the limit of detection and enrichment factor of the method were 0.5 μg L^−1^ and 200, respectively. The calibration curve was linear in the concentration range of 2–250 μg L^−1^. The relative standard deviation for six replicate experiments at 200 μg L^−1^ concentration was less than 3%. The relative recoveries of spiked urine samples were 96.3%, 101.7% and 97.3% at three different concentration levels of 50, 200 and 1000 μg L^−1^, respectively.

**Conclusion:**

DLLME procedure was successfully developed for the extraction of malathion from human urine samples. Compared to other extraction techniques, the proposed procedure had some advantages such as shorter extraction time, better reproducibility, and higher enrichment factor.

## Introduction

Pesticides are used in agriculture to destroy variety of pests that are harmful to crops. They play a critical role in supplying high-quality agricultural products. On the other hand, pesticide residues in crops can affect humane health and have unwanted side effects on the environment (1–3).

The existence of organophosphorus pesticide (OPP) residues in foods and vegetables is hazardous for human health. Some of OPP have high acute toxicity for humans. They can inhibit cholinesterase activity that causes neural impulse transmission prevention (4–6). Malathion is an OPP that is a neurotoxin and in comparison with other OPP has a lower level of toxicity to humans. Exposure to malathion can occur in different ways: oral, dermal, inhalation, or eye contact. Inhalation exposure occurs where malathion is sprayed in agricultural field or in indoor places when there is no appropriate ventilation (7–10). Malathion is quickly metabolized in the body and is not accumulated. Malaoxon is considered as its metabolite more toxic compared to the parent compound (11–13).

Due to the wide use of pesticides, there is a need for fast and reliable methods for their detection in different samples including occupational and environmental samples as well as food, fruit, and vegetable matrices. High-performance liquid chromatography (HPLC) is an appropriate choice for analysis of OPP residues in various sample matrices such as environmental water (14–16), urine, and fruits (17–20).

Sample preparation is one of the most important steps in an analytical process. This step, as an extraction procedure, results in the separation and enrichment of components from a sample matrix. Extraction procedures are common in terms of selectivity, speed, and convenience. They are varied by the conditions used and geometric configurations of the extraction phase (21).

In the past, two extraction procedures including liquid-liquid extraction (LLE) (22) and solid-phase extraction (SPE) (23, 24) were often applied for extraction and pre-concentration of analytes from sample matrices. Due to environmental consideration, recently, the miniaturized methods have attracted attention because of reduction in consumption of organic solvents by these methods. For example, in liquid-phase micro-extraction (LPME) (25) method, analytes from an aqueous sample are extracted into a small amount of a water-immiscible solvent (26–28).

Three methods named: single-drop micro-extraction (SDME), hollow fiber liquid-phase micro-extraction (HF–LPME), and dispersive liquid-liquid microextraction (DLLME) are considered as subset of the LPME method (29).

The method of DLLME was introduced at first by Rezaee et al. (2). These researchers developed a rapid, economical, and environmentally friendly sample preparation technique. The DLLME can apply for many matrices, such as soil, urine, and foodstuffs. The extraction mechanism is based on the different inclinations of the analytes to the aqueous sample and the organic extractant. The major advantages of this method are simplicity, at least use of hazardous solvents, rapid extraction, and low cost.

The aim of this study was to optimize DLLME as a quick, easy, low-cost, effective, and safe extraction technique to measure trace amounts of malathion in urine samples. The optimization important parameters affecting the efficiency of the method was performed to reach very high enrichment factor in the extraction and pre-concentration of very few malathion residue in matrix of interest.

## Materials and Methods

### Reagents and solutions

**Fig. 1:**
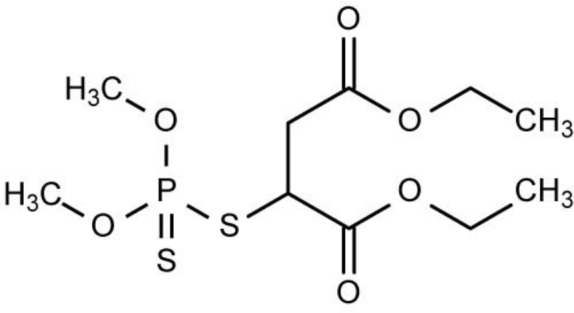
Malathion structure

The malathion (with purity >98%) were provided by Dr. Ehrenstorfer Company (Augsburg, Germany) and its structure is shown in Fig. 1. Organic solvents including carbon tetrachloride, carbon disulfide, chloroform, methanol, acetonitrile, and acetone were purchased from Merck (Darmstadt, Germany). Analytical-reagent grade sodium chloride, hydrochloric acid, and sodium hydroxide were also obtained from Merck, Germany. Deionized water was purchased from Behan Company (Tehran, Iran). A stock solution of malathion (1000 ppm) was prepared by dissolving an appropriate amount of the pesticide in acetonitrile. Working standard solutions were prepared daily by diluting the stock solution with deionized water. This study was done in 2017 at Tehran University of Medical Sciences, Tehran, Iran.

### Instrumentation

HPLC (HPLC pump k-1001, UV detector k-2600; Knauer, Germany) equipped with a UV detector was used for the separation and determination of malathion. The separation was performed on Agilent Eclipse Plus C18 column (L=250 mm, ID=4.6 mm; Reprosil-PUR C-18 AQ 10 μm) using acetonitrile-water solution (60:40, v/v) as mobile phase. The pump flowrate and column temperature were set at 1.5 mL/min and 25 °C, respectively. Under the detection wavelength of 230 nm, the chromatographic response for analytes and matrix interference was suitable. A Hettich zentrifugen Rotofix 32 (Baoding, China) was used for centrifugation. The samples were ultrasonically irradiated in a water bath at 150 W and 40 kHz using ultrasonic equipment (SonoSwiss SW 6 H). All glassware used in the experiments were washed with acetone and deionized water and then dried in an oven at 50 °C temperature.

### Dispersive liquid–liquid microextraction procedure

**Fig. 2:**
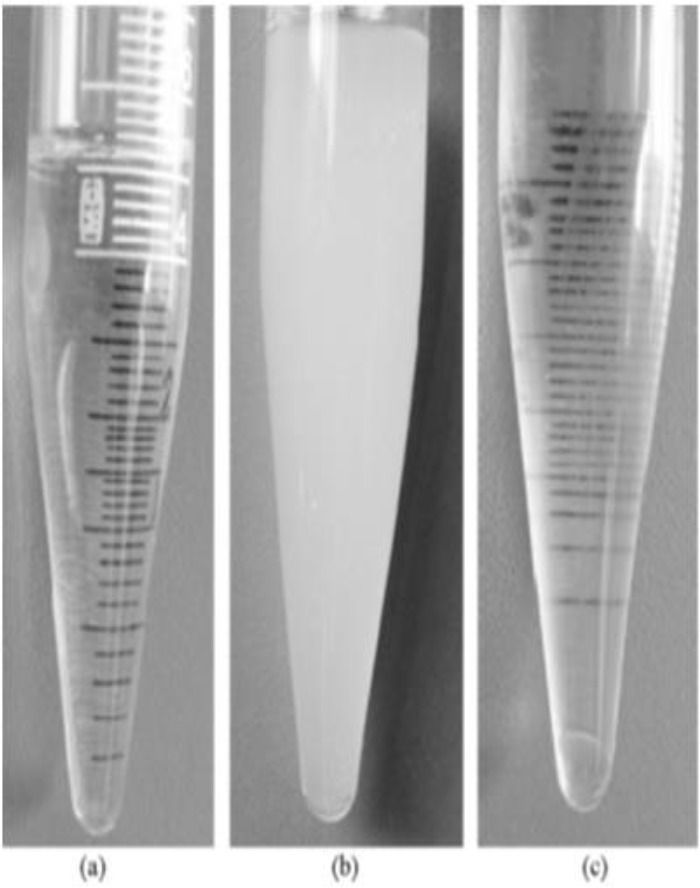
The principle of DLLME method

**Fig. 3:**
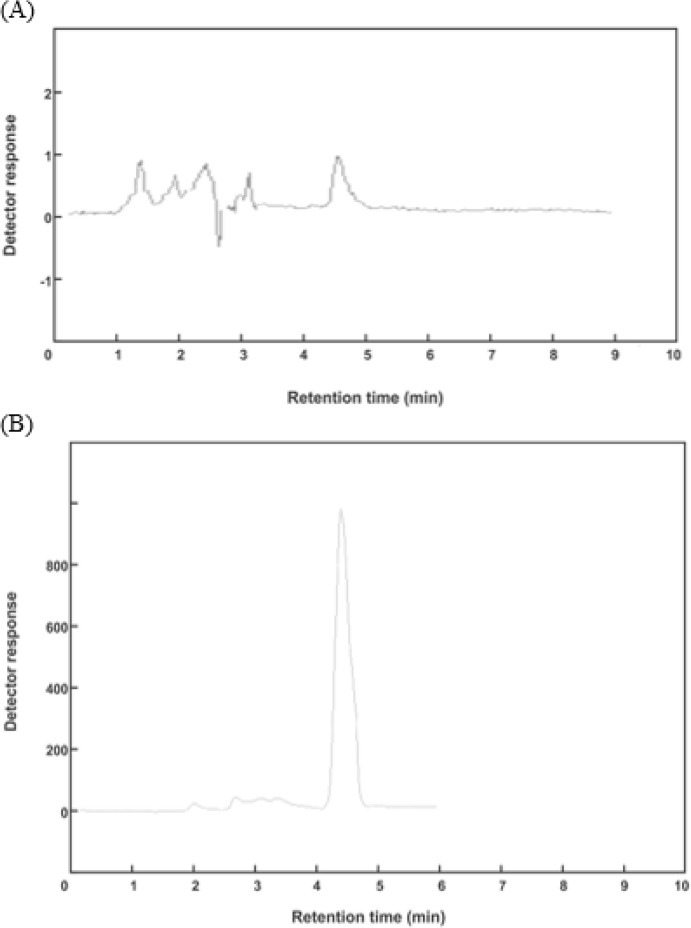
chromatograms of aqueous sample of malathion before (A) and after (B) of pre-concentration

The DLLME was performed according to the following procedures: 1) 10 mL spiked urine sample with defined concentration of analyte (1 ppm) was poured into a 15mL centrifugal tube (Fig. 2a), and 2) afterward 1.5 mL of acetonitrile containing 150 μL carbon disulfide was quickly injected to the centrifugal tube (Fig. 2b); 3) the cloudy solution was centrifuged for 5 min at 4000 rpm and the extractant were settled to the bottom of centrifugal tube (Fig. 2c); 4) The phase containing malathion was separated by a syringe and poured into another test tube, and then its solvent was evaporated under the gentle flow of N_2_ . 5) Finally, the remaining settled phase was dissolved in methanol and 20 μL of it was withdrawn using a 100 μL microsyringe and then injected into the HPLC for quantification.

In order to find the optimum factors affecting the malathion extraction, eight important factors were considered including extraction solvent, disperser solvent, volume of the extraction solvent, volume of the disperser solvent, centrifugation time and speed, salt addition, and sample pH. In each step, seven factors were constant and one factor was varied in different levels to determine the optimum quantity. Table 1 shows variables and levels of experimental design. In all experiments, malathion with concentration of 1 ppm was extracted from 10 ml sample solution. Figure 3 shows chromatograms of aqueous sample of malathion equivalent to 1 ppm before (A) and after (B) of applying DLLME procedure.

**Table 1:** Variables and levels of experimental design

***Variables***	***Levels/Types***				
Disperser solvents	Acetonitrile	Methanol	Ethanol	Acetone	-
Volume of Disperser solvent (ml)	0.5	1	1.5	2	2.5
Extraction solvent	CCl_4_	CHCl_3_	CS_2_	-	-
Volume of Extraction solvent (μl)	50	100	150	200	-
Type of shaking	manual	incubator	ultrasound	-	-
Centrifugation time (min)	5	10	15	20	-
Centrifugation speed (rpm)	2500	3000	3500	4000	
Ionic strength (NaCl%, w/v)	0	2	4	6	-
Sample pH	2	4	6	8	10

### Enrichment factor and extraction recovery

To develop DLLME procedure for enrichment of malathion, some parameters controlling extraction efficiency were investigated using sample solutions with the concentration of 1 ppm of the analyte. To evaluate the extraction efficiency, enrichment factor (EF) and extraction recovery (ER) of the analyte were calculated by equations [1] and [2], respectively.

[1] $$\text{EF}={\text{C}}_{\text{sed}}/{\text{C}}_{0}$$

Where, C_sed_ and C_0_ are the concentrations of analyte in the sediment phase and in the aqueous samples before extraction, respectively.

C_sed_ was obtained from the calibration curve of direct injection of standard solutions.

[2] $$\text{ER}={\text{C}}_{\text{sed}}{\text{V}}_{\text{sed}}/{\text{C}}_{0}{\text{V}}_{\text{aq}}\times 100\%=\text{EF}\times ({\text{V}}_{\text{sed}}/{\text{V}}_{\text{aq}})\times 100\%$$

Where, V_sed_ and V_aq_ are the volumes of the sediment phase and the aqueous sample, respectively. The average of three replicate extractions was reported for all experiments.

### Urine sample preparation

Urine specimens were collected from exposed workers, then stored in a freezer at −18 °C. Urine samples (5.0 ml) were placed in centrifuge tubes and then, the samples were diluted with 50 ml double-distilled water. The PH was adjusted at 6 with adding sodium hydroxide solutions to the samples. After that, the prepared specimen was analyzed according to the proposed preparation method.

## Results

### Selection of extraction solvent

An extraction solvent with appropriate water immiscibility and higher density than water was used in a DLLME procedure. The extraction solvent was chosen, based on its ability, to extract the analyte with good chromatogram. Three solvents including carbon tetrachloride (CCl_4_), carbon disulfide (CS_2_), and chloroform (CHCL_3_) were examined as extraction solvents in DLLME. They were selected based on important properties such as density and solubility, which could affect the extraction recovery of the target analyte. For this purpose, 10 mL aqueous solutions of malathion in defined concentration (1 ppm) were used to optimize the extraction solvent. No distinct cloudy solution was formed using CCl_4_ and CHCL_3_ as extraction solvents, meant they were not effectively dispersed among aqueous sample solution due to low extraction capability of the analyte. Extraction recoveries were the same for CCl_4_ and CHCL_3_, however, CS_2_ resulted in the highest extraction recovery for malathion. Hence, CS_2_ was selected as the optimum extraction solvent for subsequent experiments.

### Selection of disperser solvent

The type of disperser solvents is very important for obtaining preconcentration of analyte. The solvents have been chosen must be appropriately miscible in both extraction solvent and sample solution, so that, they can form a distinct cloudy solution. Due to such consequence, four possible disperser solvents including methanol, ethanol, acetonitrile, and acetone were tested. Acetonitrile displays the highest extraction recovery for the analyte in comparison with the other mentioned solvents. Therefore, acetonitrile was selected for later experiments.

### Effect of volume of extraction solvent

To evaluate the effect of extraction solvent volume on the enrichment factor and extraction recovery of analyte, the experiments were performed by using 2 mL acetonitrile containing different volumes of CS_2_ (50, 100, 150 and 200 μL). The extraction recoveries of malathion increases when the volume of CS_2_ raises. The volume of the sediment phase at the bottom of the test tube was increased by increasing the volume of CS_2_ from 50 to 150 μL. At more volume of 150 μL of CS_2_, the most extraction recovery was achieved and after that it was constant. Therefore, the volume of 150 μL was selected as the optimal volume of CS_2_.

### Effect of volume of disperser solvent

The other parameter that had effect on the extraction recovery and enrichment factor was the volume of disperser solvent. Different acetonitrile volumes (0.5, 1, 1.5, 2, 2.5 mL) containing 150 μL CS_2_ were performed to reach the optimal volume. The extraction recoveries of the analyte was increased at first, then, it was decreased as the volume of acetonitrile was being increased. At low volumes of acetonitrile, the cloudy solution was not formed completely, so the extraction recovery of analyte was low, however, at high volume of acetonitrile up to 2 mL, the solubility of CS_2_ in aqueous solution was decreased, then, the extraction recovery was increased. However, at the volume of more than 2 mL, the extraction recovery was decreased. Overall, 1.5 mL acetonitrile was chosen in order to obtain high extraction recovery and good enrichment factor.

### Optimization of centrifugation time and speed

Centrifugation is a critical step in DLLME to separate the extractant phase. This step destroys the cloudy solution and helps the extraction solvent to settle at the bottom of the tube. The effect of centrifugation time and speed on the extraction recovery were examined and optimized in the ranges of 5–20 min and 2500–4000 rpm, respectively. The time of 5 min and the speed of 4000 rpm were selected as the centrifugation parameters for subsequent experiments.

### Salt addition

Addition of salt increase ionic strength which can improve the extraction recovery. Salting out can decrease the solubility of the analyte in the aqueous phase and its extraction into the organic phase can be better. For examination the effect of salt addition, different concentrations of sodium chloride (0, 2, 4, and 6% w/v) were investigated. When the concentration of NaCl increased, then the viscosity of the aqueous phase increases and it causes reduction in diffusion coefficients of the analyte. Therefore, the extraction recovery decreased. Therefore, next experiments were performed in the absence of salt.

### Effects of sample pH

The sample pH is also important because the analyte formation is dependent on it and have effect on the extraction recovery. By adding the appropriate hydrocholoric acid or sodium hydroxide solutions to water samples, stability of malathion under the pH range of 2–10 were investigated. The results indicated the extraction recovery of the analyte reached a high level at pH 6. Therefore, doubled-distilled water was used without pH adjustment in the study.

### Analytical features of the method

The analytical characteristics of the method, including linear range (LR), limit of detection (LOD), limit of quantification (LOQ), correlation coefficient (r^2^), relative standard deviation (RSD%), enrichment factor (EF), and extraction recovery (ER), were determined under the optimized conditions to evaluate the performance of the method. The obtained results are summarized in Table 2.

**Table 2:** Quantitative features of the proposed method for malathion

***LR (μg L^−1^)***	***r^2^***	***MDL (μg L^−1^)***	***LOQ (μg L^−1^)***	***RSD (%) 200 μg L^−1^ spiked (n=6)***		***EF***	***ER (%)***
				***Intra-day***	***Inter-day***		
5–500	0.9914	1.5	5	1.4	3.03	220	91.5

Linearity is over a broad concentration range for the pesticide, with correlation coefficients (r) 0.991. The MDL (LOD) and LOQ, calculated on the basis of signal to noise ratio (S/N) of 3 and 10, were 0.5 and 5, respectively. The RSD values are less than 4% for inter-day and intra-day precision, indicating acceptable repeatability for the developed method. The EF and ER for the pesticide were 220 and 91.5%, respectively. Satisfactory repeatability, high EF and ER, and low MDL and LOQ are the main advantages of the proposed method.

### Validation of the optimized method

The feasibility of the proposed method was applied to the preconcentration and determination of malathion from spiked urine sample. In order to validate the accuracy of the DLLME procedure, samples were spiked with the target analyte at three different concentration levels of 50, 200, and 1000 μg L^−1^ and then analyzed in triplicate using the recommended method. The analyte recoveries are shown in Table 3. The relative recoveries are in the range of 96.3%–101.7%. The relative recoveries of the analyte did not vary significantly at different spiking concentration levels of 50, 200 and 1000 μg L^−1^. The values of recoveries confirm the validity of the proposed method. The obtained RSDs for the real samples were fairly low at different concentrations. The real sample matrix has a little effect on the proposed method for pre-concentration of malathion from urine sample.

**Table 3:** Relative recovery (RR) and RSD values of malathion in urine sample

***Spiked levels (μg L^−1^)***	***RR (%) (n=3)***		***RSD (%) (n=3)***	
	***Intra-day***	***Inter-day***	***Intra-day***	***Inter-day***
50	96.3 ± 1.5^a^	92.5 ± 2.5^a^	1.5	2.5
200	101.7 ± 3.1	97.7 ± 3.4	3	3.5
1000	97.3 ± 1.5	102.5 ± 2.8	1.6	3

a Mean of three determinations ± standard deviation

## Discussion

The comparison of LR, RSD, LOD, and LOQ obtained for the presented method with those of other reported methods for analysis of the target analyte in different samples is summarized in Table 4. The RSD of the proposed method is comparable and better than those reported for the other methods. This study presented low and acceptable MDL (LOD) and LOQ compared to the most reported methods. Most of the mentioned techniques have been used a high selective or sensitive detection systems, such as flame photometric detection (FPD), flame ionization detector (FID) or mass spectrometry (MS). It must take into account that MS, FID, and FPD detectors in combination with gas chromatography (GC) and ultra-pressure liquid chromatographic (UPLC) are expensive and cannot be used widely for the analysis of organophosphorus pesticides in developing countries. However, in the present work has used no special detectors. Therefore, the developed method is sensitive, simple, rapid, and repeatable and it can be used for the extraction and pre-concentration of malathion residues from aqueous samples.

**Table 4:** Comparison of the presented method with other methods used in the analysis of the target analyte

***Pesticide***	***Sample***	***LOD (μg L^−1^)***	***LOQ (μg L^−1^)***	***RSD (%)***	***LR (μg L^−1^)***	***Extraction Factor***	***Extraction time (min)***	***Method***	***Reference***
Malathion	Aqueous sample	0.28	-	2.1	0.5–100	50	30	SPME–GC–FPD	(30)
Diazinon	Water	1.4	-	9.4	5–500	-	20	PN–SDME–GC–MS	(31)
Chlorpyrifos	sample	1.6		8.3	5–500				
Malathion		1.6		7	5–500				
Diazinon	Aqueous	0.65	2.2	6	3–40000	-	15	MWA–DLLME–GC–	(32)
Chlorpyrifos	sample	0.74	2.5	4	3–40000			FID	
Malathion		1.3	4.5	4	3–40000				
Malathion	Soil & water sample	0.026 × 10^−3^	0.078 ×10^−3^	4.9	10–1500	-	0.6	SPE-UPLC-MS	(33)
Malathion	Urine	1.5	5	3.03	5–500	>200	5	DLLME–HPLC–UV	This work

## Conclusion

DLLME procedure was successfully developed for the extraction of malathion from urine samples. Different conditions for the extraction of the analyte were investigated and optimized. Compared to the other extraction techniques, the proposed optimized procedure had more advantages such as shorter extraction time, better reproducibility, and higher enrichment factor. The analytical figures of merit such as good precision and enrichment factor, suitable recoveries, broad dynamic linear range, and low limit of detection were attained due to DLLME method. The proposed method can be used as a simple procedure to yield high preconcentration efficiency for determination of malathion in complex matrices.

## Ethical considerations

Ethical issues (Including plagiarism, informed consent, misconduct, data fabrication and/or falsification, double publication and/or submission, redundancy, etc.) have been completely observed by the authors.
